# Persistent SARS‐CoV‐2 infection in patients seemingly recovered from COVID‐19

**DOI:** 10.1002/path.6035

**Published:** 2023-01-18

**Authors:** Rossana Bussani, Lorena Zentilin, Ricardo Correa, Andrea Colliva, Furio Silvestri, Serena Zacchigna, Chiara Collesi, Mauro Giacca

**Affiliations:** ^1^ Department of Medical, Surgical and Health Sciences University of Trieste Trieste Italy; ^2^ International Centre for Genetic Engineering and Biotechnology (ICGEB) Trieste Italy; ^3^ School of Cardiovascular Medicine & Sciences King's College London, British Heart Foundation Centre of Research Excellence London UK

**Keywords:** COVID‐19, post‐mortem analysis, SARS‐CoV‐2, syncytia, spike, nucleocapsid, parabronchial glands, cartilage

## Abstract

SARS‐CoV‐2 infection is clinically heterogeneous, ranging from asymptomatic to deadly. A few patients with COVID‐19 appear to recover from acute viral infection but nevertheless progress in their disease and eventually die, despite persistent negativity at molecular tests for SARS‐CoV‐2 RNA. Here, we performed post‐mortem analyses in 27 consecutive patients who had apparently recovered from COVID‐19 but had progressively worsened in their clinical conditions despite repeated viral negativity in nasopharyngeal swabs or bronchioalveolar lavage for 11–300 consecutive days (average: 105.5 days). Three of these patients remained PCR‐negative for over 9 months. Post‐mortem analysis revealed evidence of diffuse or focal interstitial pneumonia in 23/27 (81%) patients, accompanied by extensive fibrotic substitution in 13 cases (47%). Despite apparent virological remission, lung pathology was similar to that observed in acute COVID‐19 individuals, including micro‐ and macro‐vascular thrombosis (67% of cases), vasculitis (24%), squamous metaplasia of the respiratory epithelium (30%), frequent cytological abnormalities and syncytia (67%), and the presence of dysmorphic features in the bronchial cartilage (44%). Consistent with molecular test negativity, SARS‐CoV‐2 antigens were not detected in the respiratory epithelium. In contrast, antibodies against both spike and nucleocapsid revealed the frequent (70%) infection of bronchial cartilage chondrocytes and para‐bronchial gland epithelial cells. In a few patients (19%), we also detected positivity in vascular pericytes and endothelial cells. Quantitative RT‐PCR amplification in tissue lysates confirmed the presence of viral RNA. Together, these findings indicate that SARS‐CoV‐2 infection can persist significantly longer than suggested by standard PCR‐negative tests, with specific infection of specific cell types in the lung. Whether these persistently infected cells also play a pathogenic role in long COVID remains to be addressed. © 2023 The Authors. *The Journal of Pathology* published by John Wiley & Sons Ltd on behalf of The Pathological Society of Great Britain and Ireland.

## Introduction

Two years after the pandemic, it has now become clear that SARS‐CoV‐2 infection can result in highly heterogeneous disease. COVID‐19 ranges from asymptomatic to deadly, with variable severity according to age, concomitant conditions, and ethnicity [1, 2, 3]. Our previous work based on pathological examination of lungs from COVID‐19 patients who succumbed to the disease has shown extensive alveolar damage, thrombosis of both micro‐ and macro‐vasculature, and long‐term persistence of viral RNA in pneumocytes and endothelial cells [4]. In almost 90% of patients, we detected a large number of dysmorphic pneumocytes, forming syncytial elements as a consequence of the fusogenic activity of the SARS‐CoV‐2 spike protein [5].

The course of sign and symptom resolution in cases with moderate or severe disease who recover from the acute phase appears equally variegate. Recovery is prompt and relatively fast in most patients. A recent histopathological examination of elective lung resections to analyse the changes in these COVID‐19 survivors identified no differences between the lung parenchyma of these patients to that of controls, in contrast to the acutely infected cases [6]. Epidemiological studies, however, indicate that over one third of patients do not recover completely at 14–21 days post‐infection, and some of them remain symptomatic for several months [7, 8, 9, 10]. Most patients affected by post‐COVID syndrome (commonly called ‘long COVID’) become PCR‐negative, indicating apparent elimination of the virus.

Here, we focus our attention on a third category of patients, who apparently clear the virus but nevertheless progress into their disease and eventually die. We analysed a cohort of consecutive patients who resulted negative at SARS‐CoV‐2 PCR tests for up to 300 days after remission from acute infection, despite progressive worsening of their clinical status and eventual death. The aim of this study was to study the pathological correlates of this condition, including the possible persistence of viral infection. Post‐mortem analyses of these patients revealed the frequent, long‐term presence of virus‐infected cells in specific lung structures, including bronchial glands and cartilage. Thus, the progressive worsening of clinical conditions in apparently PCR‐negative patients after COVID‐19 is often associated with the persistent infection of specific cell types in the lung.

## Materials and methods

### Case cohort

Post‐mortem analysis was performed at the Pathology Unit of the University Hospital and School of Medicine in Trieste, Italy. Since the beginning of the pandemic in early 2020, the Pathology Unit has performed over 800 full body autopsies in COVID‐19 patients, including analysis of lung, brain, heart, and kidney tissues. The same pathologist (RB) analysed all cases in this study, thus excluding operator‐dependent biases. Clinical data, including age, gender, known co‐morbidities, and therapies, were collected and are shown in Table 1 and supplementary material, Table S1.

**Table 1:** Selected clinical and histopathological features of the former COVID‐19 patients reported in this study.

Patient code	Age	Sex	Duration of PCR positivity (days)	Time between last negative PCR test and death (days)	Pneumonia score (0 to 5)	Fibrosis score (0 to 4)	Cartilage alterations	Syncytia	SARS‐CoV‐2 (IHC Spike/N)	SARS‐CoV‐2 Orf1ab (number of copies)
**AUT. 256.20**	99	F	9	11	4*	1		+	+++	‐
**AUT. 314.20**	89	F	37	16	4*	1		+	−	‐
**AUT. 327.20**	75	F	5	43	4*	2		+	−	‐
**AUT. 448.20**	72	M	25	81	3*	1		+	+	181.2
**AUT. 560.20**	71	M	31	164^†^	5^‡^	5	Severe	+	+++	518.3
**AUT. 614.20**	79	M	16	155	3*	1		+	−	‐
**AUT. 650.20**	82	F	32	110	1	2			+	
**AUT. 692.20**	86	F	25	186	2*	1			++	
**AUT. 780.20**	61	F	53	197	3*	2	Moderate	+	+++	‐
**AUT. 830.20**	77	F	67	216	3*	2	Severe	+	++	220
**AUT. 831.20**	91	F	24	201	2	2	Mild		+	48.2
**AUT. 809.20**	80	M	15	16	3*	3		+	−	19.7
**AUT. 823.20**	73	F	25	10	3*	2		+	++	
**AUT. 850.20**	68	M	17	16	5^‡^	4	Moderate	+	++	
**AUT. 857.20**	86	M	73	218^†^	1	2	Mild		−	11.9
**AUT. 41.21**	74	M	10	35^†^	5^‡^	3	Moderate	+	+	
**AUT. 52.21**	73	M	13	281	4*	3	Moderate	+	+	‐
**AUT. 108.21**	72	F	12	30^†^	5^‡^	3	Mild	+	++	5050.8
**AUT. 144.21**	90	F	83	21	1	2		+	−	700.6
**AUT. 145.21**	62	F	46	18	5^‡^	4		+	+++	‐
**AUT. 165.21**	78	M	13	78^†^	5^‡^	4	Severe	+	−	‐
**AUT. 183.21**	52	F	37	14^†^	5^‡^	4	Mild	+	+++	2583.6
**AUT. 208.21**	72	F	14	300^†^	3*	3		+	−	
**AUT. 223.21**	95	M	28	270^†^	3	2		+	+	
**AUT. 237.21**	53	M	9	56^†^	5^‡^	4		+	+	
**AUT. 299.21**	76	M	28	21	5^‡^	4	Mild	+	+	
**AUT. 315.21**	77	F	15	84^†^	2*	3			++	

* Pneumonia as the primary cause of death;

^†^ Molecular test also performed in BAL;

^‡^ Pneumonia as a joint cause of death.

### Diagnostic PCR test

Combined oropharyngeal and nasopharyngeal swabs were collected for RNA extraction, followed by SARS‐CoV‐2 genome quantitative real‐time PCR quantification using the Liferiver Novel Coronavirus (2019‐nCoV) Real Time Multiplex RT‐PCR Kit (#ZJ0010; Liferiver Bio‐Tech, San Diego, CA, USA). This assay measures simultaneously three target genes in a single tube: SARS‐CoV‐2 gene E, gene N, and gene ORF1ab, and it includes a positive control and an internal control, quoting an analytical sensitivity of 1 × 10^3^ copies/ml for both combined oropharyngeal and nasopharyngeal swabs and bronchioalveolar lavage.

### Ethics

This study was approved by the Joint Ethics Committee of the Regione Friuli Venezia Giulia, Italy (re. 0019072/P/GEN/ARCS).

### Histological analysis

Samples were fixed in 10% formalin for at least 50 h and then embedded in paraffin. One‐micrometre sections were deparaffinised in xylene, rehydrated, and processed for haematoxylin and eosin or immunohistochemical staining.

The severity of pneumonia was defined according to the following scores (from 0 to 5): 0, absence of inflammation; 1, interstitial oedema, exudative alveolar damage; 2, same as 1 plus mild alveolar epithelial exfoliative damage with mild interstitial or alveolar inflammation; 3, diffuse exfoliative alveolar damage, foci of endothelialitis and endothelial necrosis, interstitial inflammation, occasional vasculitis; 4, marked interstitial, alveolar, and perivascular inflammation, necrotic exfoliation of alveolar epithelium and vascular endothelium, reactive pneumocyte hyperplasia, presence of endoalveolar macrophages, microvascular damage; and 5, same as 4 but extended to all analysed samples, reduced functional lung parenchyma, stage II or III acute respiratory distress syndrome (ARDS), with possible bacterial or fungal superinfections.

The extent of lung fibrosis was defined according to the following scores (from 0 to 4): 0, absence of fibrosis; 1, occasional foci of recent, poorly structured interstitial fibrosis; 2, small foci of recent, poorly structured fibrosis together with areas of more structured, matured (hyalin) fibrosis; 3, multifocal unstructured and hyalin fibrosis, often endoalveolar; and 4, large areas of polychronic fibrosis with disorganisation of the interstitial space and the alveolar structures.

Cartilage alterations were classified as mild (structural modifications of chondrocytes), moderate (abnormal chondrocytes, with perinuclear halos and altered intracellular organisation), or severe (important morphological alterations of chondrocytes, with signs of structural disorganisation and necrosis).

### Immunohistochemistry

Antigen retrieval was performed in boiling sodium citrate solution (0.01 m, pH 6.0; 0.05% Tween‐20) for 20 min. Sections were allowed to cool and permeabilised for 10 min in 1% Triton X‐100 in PBS. Prior to blocking for the primary antibody, endogenous biotin was suppressed using the Endogenous Biotin‐Blocking Kit (E21390; Thermo Fisher Scientific, Waltham, MA, USA) according to the manufacturer's instructions. Samples were then blocked in 2% BSA (Roche, Burlington, MA, USA) and incubated overnight at 4 °C with the following primary antibodies diluted in blocking solution: SARS‐CoV‐2 spike protein (GTX632604 [1A9]; Genentech, South San Francisco, CA, USA; 1:250 [11, 12]), SARS‐CoV‐2 nucleocapsid protein (40143‐R001; Sino Biological, Wayne, PA, USA; 1:150 [13]), ACE2 (ab15348; Abcam, Cambridge, MA, USA), and TMEM16F (HPA038958; Sigma‐Aldrich, St Louis, MO, USA). After endogenous peroxidase inhibition with 3% H_2_O_2_ for 10 min at room temperature, sections were incubated with the appropriate biotin‐conjugated secondary antibody for 1 h at room temperature.

Following signal amplification with avidin–biotin‐complex–HRP (Vectastain; Vector Laboratories, Burlingame, CA, USA), DAB solution (Vector Laboratories) was applied for 2–3 min. Haematoxylin (Bioptica, Cambridge, UK) was further used to stain nuclei and Bluing reagent was used on a Ventana (Roche) automated staining system. Images were acquired using a Leica ICC50W light microscope (Leica Microsystems, Wetzlar, Germany).

Post‐mortem analysis of PCR‐positive, COVID‐19 patients and pre‐2019 negative controls to ensure specificity has previously been demonstrated [4, 5].

### RNA extraction and RT‐qPCR detection of SARS‐CoV‐2 RNA

Post‐mortem sample collection dates back to late 2020–early 2021 when the prevalent circulating variant was B.1.617.2 (Delta), with no known diffusion of other variants, and before the vaccination campaign took off in Italy – none of the patients in this study were vaccinated. The presence of SARS‐CoV‐2 RNA was detected by RT‐qPCR in formaldehyde‐fixed, paraffin‐embedded tissues. RNA extraction was performed from 8 μm sections which were first deparaffinised twice using 1 ml of xylene and centrifuged at 12,000 × *g* for 5 min. Then samples were washed twice with 1 ml of absolute ethanol and centrifuged at 12,000 × *g* for 5 min. Tissues were then digested with tissue lysis buffer [20 mm Tris–HCl (pH 8.0), 1 mm CaCl_2_, 0.1% sodium dodecyl sulphate, and 200 μg/ml proteinase K] and incubated at 50 °C overnight under constant shaking (1,500 rpm, Eppendorf ThermoMixer; Eppendorf AG, Hamburg, Germany). Next, RNA extraction was performed with 1 ml of Trizol (Invitrogen, brand of Thermo Fisher Scientific, Waltham, MA, USA), according to the manufacturer's instructions.

The quality of total RNA was assessed by measuring the expression of GAPDH by qRT‐PCR. A GAPDH Ct value ≤35 was considered acceptable. First, cDNA was synthesised using 2 μg of total RNA using the Thermo Scientific First Strand cDNA Synthesis Kit (Thermo Fisher Scientific), following the manufacturer's instructions. Samples were then diluted 1:4 and qRT‐PCR was performed using the GoTaq® qPCR Master Mix Kit (Promega, Madison, WI, USA) in a CFX96 Touch Real‐Time PCR Detection System (Bio‐Rad, Hercules, CA, USA). A known positive formaldehyde‐fixed, paraffin‐embedded sample, which was subjected to the same RNA extraction procedure, was used as a positive control. Of note, post‐mortem sample collection could only be performed 24–48 h after death, which could have resulted in RNA degradation in some of the samples.

The detection of SARS‐CoV‐2 was performed using the Luna Universal Probe One‐Step RT‐qPCR Kit (New England Biolabs, Ipswich, MA, USA) using primers and probes (Eurofins Genomics, Ebersberg, Germany) for N and ORF1ab genes that have previously been reported [14].

## Results

We performed post‐mortem analyses in 27 consecutive patients who died at the University Hospital in Trieste (Italy) with the characteristics of having had recent SARS‐CoV‐2 infection followed by apparent virological remission for at least 10 days. The enrolment period was from 1 April 2020 to 31 January 2021. The main features of these patients are reported in Table 1. The average age of this population was 76.4 years; 56% were females. All individuals had a history of COVID‐19 infection with different severities, with a first period of PCR‐positive swab tests for 5–83 consecutive days (mean: 28.2 days) followed by a period of apparent clearance from infection lasting from 11 to 300 consecutive days (average: 105.5 days) before death. A PCR test was performed every 3 days on all hospitalised patients. During this apparent remission, multiple nasopharyngeal swabs or bronchioalveolar lavage RT‐PCR tests were consistently negative for viral RNA.

Three of these patients remained PCR‐negative for over 9 months and had at least ten tests performed in this period. Despite this apparent SARS‐CoV‐2 negativity, all of these patients either progressed in their pulmonary disease or required hospitalisation for other causes and eventually died. Clinically, pneumonia was either the single or the joint primary cause of death in most cases (22/27, 81.5%). Additional information on the clinical history and medications taken during hospitalisation is reported in supplementary material, Table S1. Only patients in the ICU were administered corticosteroids. There was no apparent correlation between any of the drugs administered and viral persistence.

### Post‐mortem analysis of PCR‐negative, former COVID‐19 patients

For each patient, we studied at least ten different parenchymal samples from each lung, plus an additional four samples in proximity to the pulmonary hilum, to include the bronchial cartilage. Each of the patients had an average of 24 independent specimens investigated. For each sample, two inclusions were selected and from each of these inclusions, at least ten sections were prepared (over 600 sections in total).

**Figure 1:**
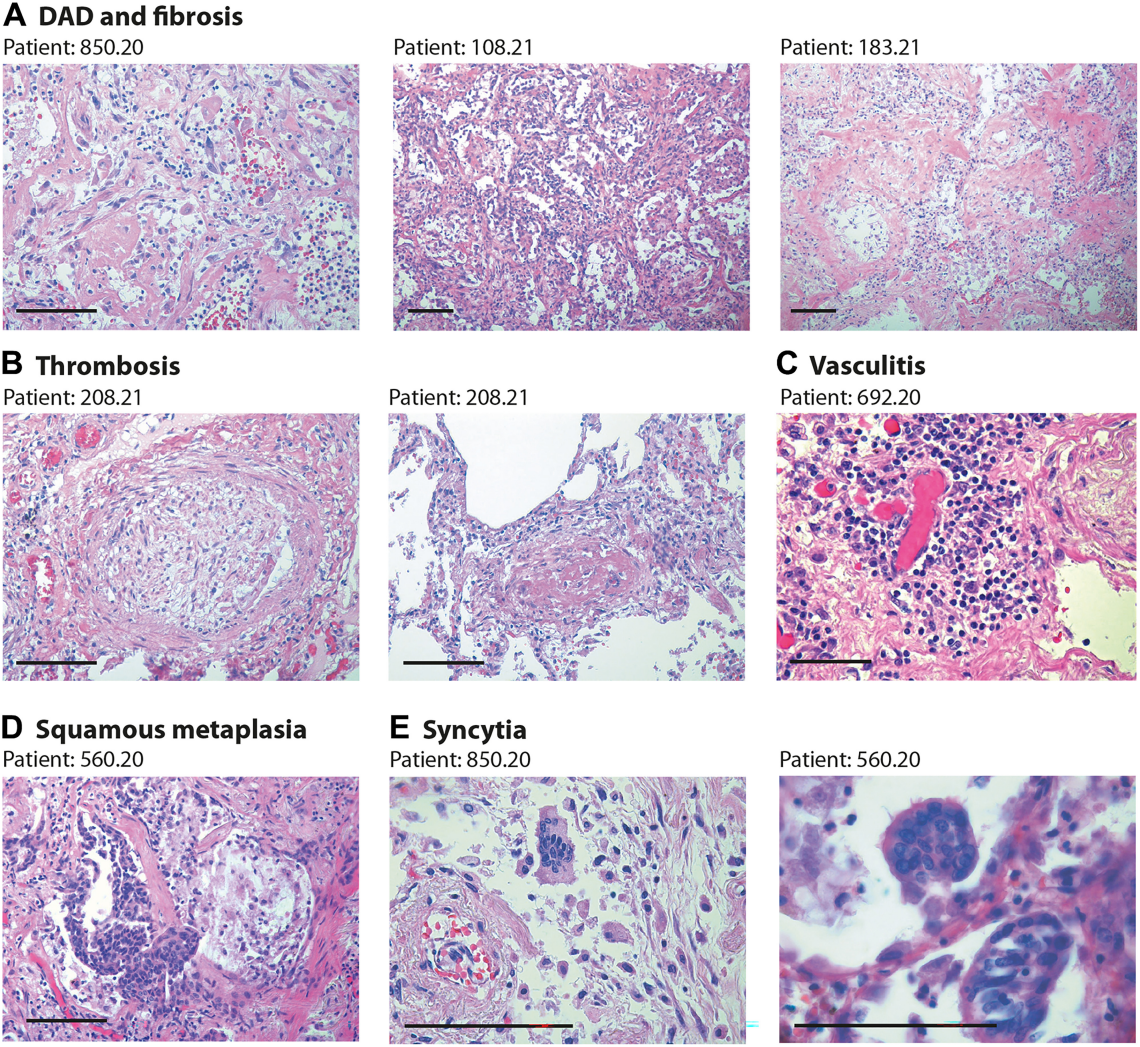
Lung pathology in former COVID‐19 patients. Haematoxylin and eosin (H&E) staining showing characteristic pathological features of previous COVID patients (patient's code is at the top of each panel). Scale bars in all panels: 100 μm. (A) Diffuse alveolar damage (DAD) and parenchymal fibrosis. The pictures, taken from three different patients, show massive alveolar inflammation with almost complete occlusion of the alveolar lumen; delamination of pneumocytes, which appear grossly damaged and dystrophic; endoalveolar fibrin deposition; and fibrosis of the inter‐alveolar septa, which is massive in patient 183.21. (B) Thrombosis of a medium‐ (left) and small‐ (right) sized artery. The thrombotic artery on the left is surrounded by hyalin fibrotic tissue and flanked by congested small vessels. Thrombi are in different stages of organisation. (C) Perivascular inflammatory cap in a small thrombotic vessel. (D) Squamous metaplasia (pseudosyncytia) of the respiratory epithelium. Massive defoliation of the metaplasic alveolar cells, only a few of which maintain a cylindrical ciliated appearance. Massive congestion of the surrounding small vessels. (E) Presence of syncytial cells in two patients. In the upper panel, the syncytium is in the context of a delaminated alveolar structure and in proximity to an arteriole with a thickened wall and exfoliated endothelium.

All patients but five (81%) had histological evidence of diffuse or focal interstitial pneumonia, also in accordance with the clinical information. Pneumonia was staged as severe in 13 (48%) cases and was considered the final cause of death. This included two of the three cases that tested PCR‐negative for over 9 months. Pneumonia was characterised by diffuse alveolar damage and immune cell infiltration, and was accompanied by extensive fibrotic substitution in 13 cases (48%; fibrotic score ≥ 3) (see Figure 1A for representative images in different patients). As for acute COVID‐19 disease [15, 16, 17, 18, 19, 20], thrombosis of the micro‐ and macro‐vasculature was a common finding (18/27 patients, 67%). Thrombi showed different stages of organisation, with some thrombi still infiltrated by inflammatory cells and others in an advanced, fibrotic stage (Figure 1B). Inflamed vessels showing a perivascular lymphocyte cap, compatible with an ongoing vasculitis [21, 22], were observed in 10/41 patients (24%); this often occurred in thrombotic vessels (Figure 1C). Cytological abnormalities were also detectable in all cases. These included squamous metaplasia of the respiratory epithelium (8/27; 30% cases; Figure 1D) and the presence of dysmorphic cells with multiple nuclei (Figure 1E). These syncytial cells were present in 18/27 (67%) patients and are one of the hallmarks of COVID‐19 lung pathology [4] as a consequence of the fusogenic activity of the SARS‐CoV‐2 spike protein [5]. Additional images from one of these patients, including immunoreactivity of perivascular cells in vasculitis, are shown in supplementary material, Figure S1. Overall, the lung pathology of these patients was not significantly different from that observed in patients succumbing to acute viral infection [4], in spite of the apparent, long‐term virological remission.

**Figure 2:**
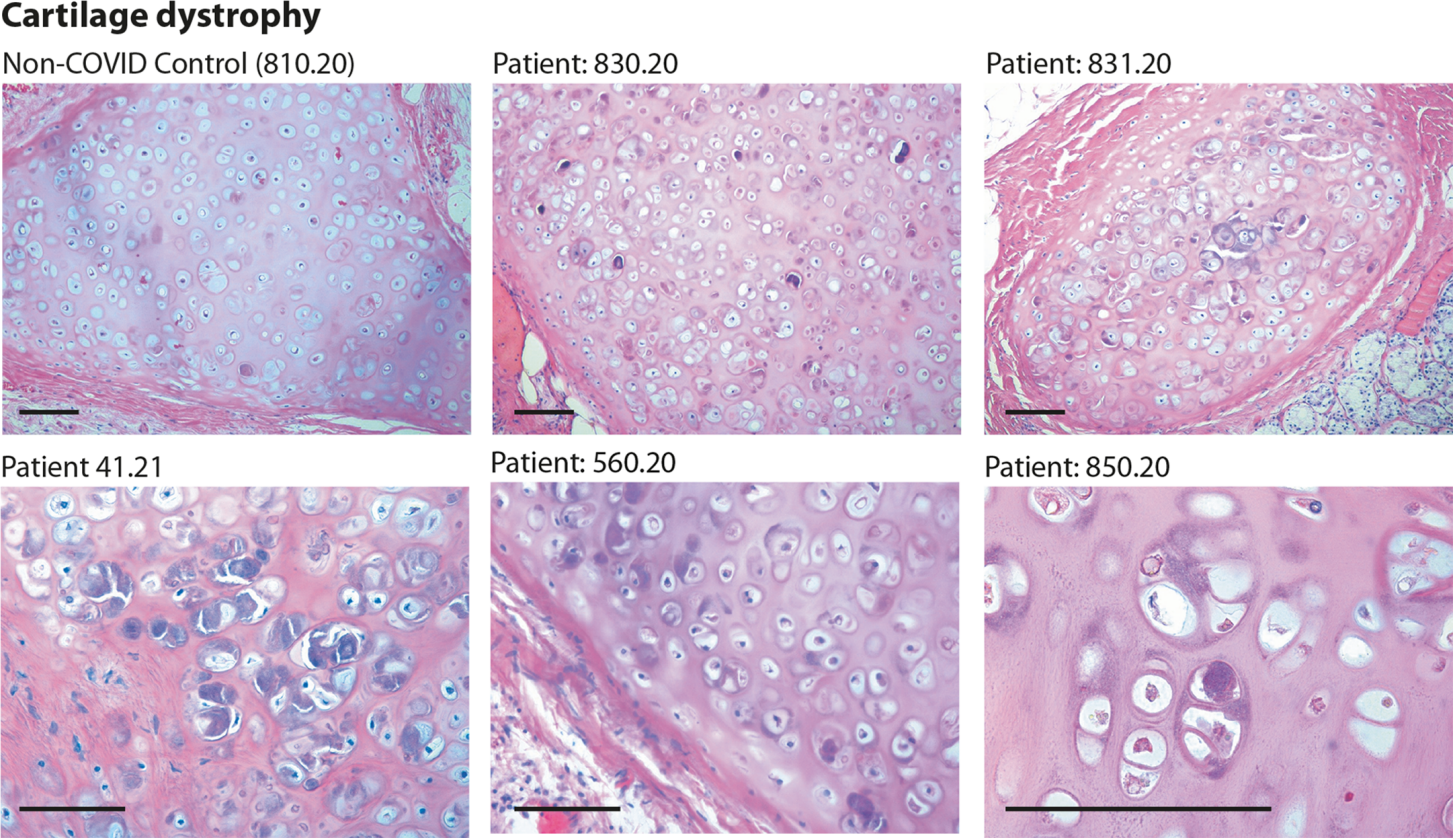
Histology of bronchial cartilage. Haematoxylin and eosin (H&E) staining is shown for five previous COVID‐19 patients and one normal control who died from non‐COVID pneumonia. In previous COVID‐19 patients, chondrocytes appear randomly clustered. Several cells show marked basophilic degeneration, with increased size, dysmorphic nuclear structure, and peripheral halos. In several instances, the chondrocytes have become anucleated. Scale bars: 100 μm.

One common characteristic of these former COVID‐19 patients in apparent remission was the presence of dysmorphic features in the bronchial cartilage. These were present in 12/27 patients (44%) with variable penetrance (Table 1). Dystrophic cartilage showed a fibrous‐hyaline degeneration of the tracheal rings, together with multiple pyknotic nuclei, consistent with necrobiosis of chondrocytes (shown in Figure 2 for patients 830.20, 831.20, 41.21, 560.20, and 850.20 at different magnifications; a normal cartilage sample from an individual who died in the same period for other causes is shown in the top‐left panel).

Collectively, these findings reveal that most patients with previous COVID‐19 who progressively deteriorated in their clinical status despite apparent molecular negativity to SARS‐CoV‐2 still show features of acute viral infection, including lung thrombosis, vasculitis, and the presence of abnormal syncytial cells (recently reviewed in [23]), in addition to cartilage dystrophy. This prompted us to investigate whether these pathological alterations could indeed be consequent to undetected, but persisting, viral infection.

### Persistence of viral infection in defined lung districts

**Figure 3:**
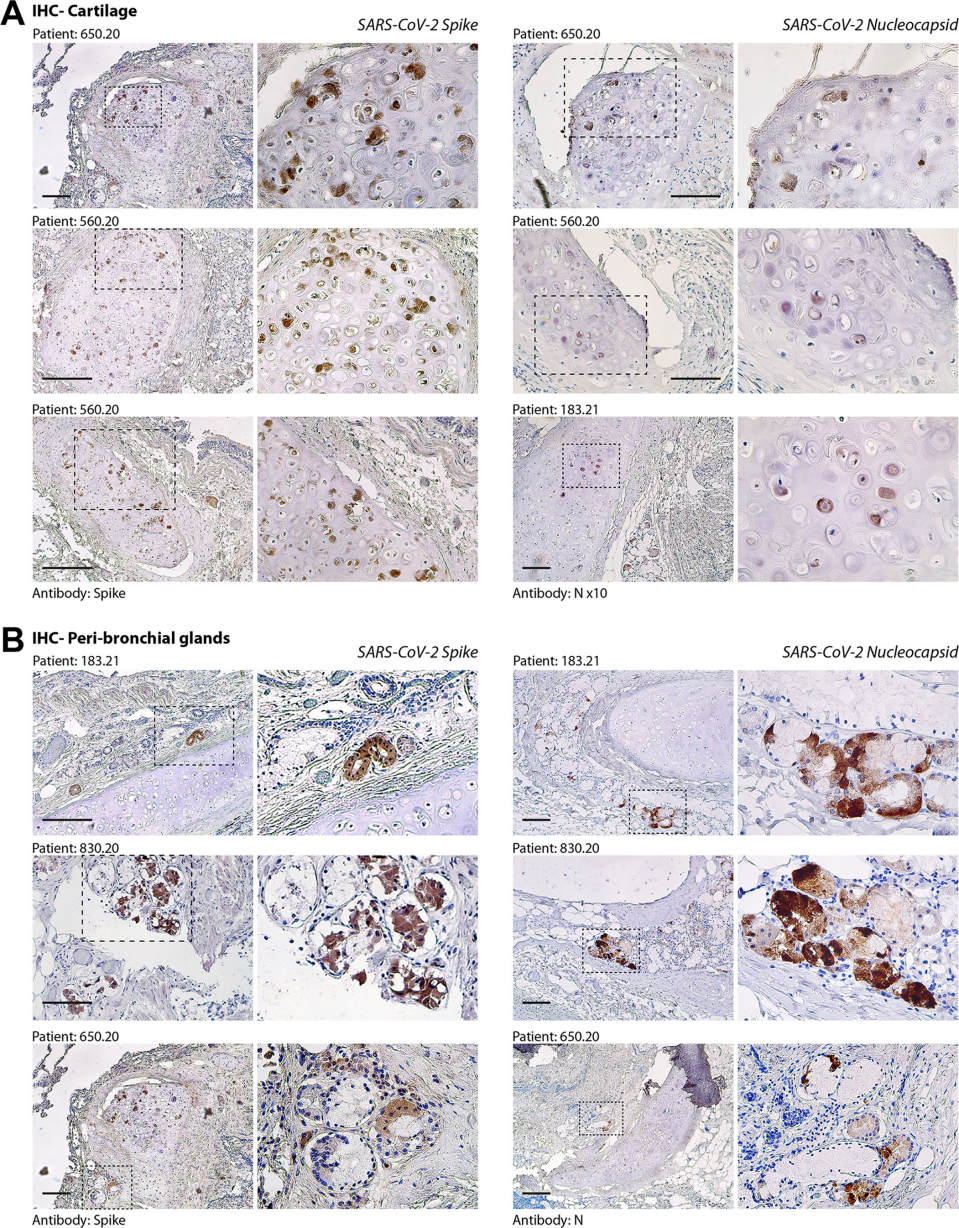
Immunohistochemistry (IHC) for SARS‐CoV‐2 spike and nucleocapsid (N) antigens. (A) Evidence of SARS‐CoV‐2 infection in bronchial cartilage chondrocytes. For each of the patients (indicated at the top of each pair of pictures), low‐ and high‐magnification pictures are shown; the magnified area is indicated by a dashed box. Most of the positive chondrocytes (in dark brown) show dysmorphic features. Scale bars in all panels: 100 μm. (B) Same as in panel A for the area surrounding the bronchial structures. Several mucosal glands proximal to the bronchial cartilage were positive for both spike and N antigens.

We investigated the presence of virus‐infected cells using two antibodies that specifically detect the spike and nucleocapsid (N) proteins of SARS‐CoV‐2 by immunohistochemistry (IHC). We have optimised IHC with these antibodies previously [4, 5]. Supplementary material, Figure S2 shows the absence of positive cells in lung samples from patients who died of COVID‐19‐unrelated conditions. In none of the patients from our cohort did we find the presence of spike‐ or nucleocapsid‐expressing cells in the respiratory epithelium (supplementary material, Figure S3). This is consistent with the absence of SARS‐CoV‐2 RNA by RT‐PCR in the diagnostic tests, including bronchoalveolar lavage. However, we detected virus‐infected cells in specific lung districts in 19/27 (70%) cases. In particular, several chondrocytes were clearly positive for the expression of both spike and nucleocapsid antigens in the bronchial cartilage (representative images at two magnifications are shown in Figure 3A, left and right panels, respectively), with most of the chondrocytes showing dysmorphic features.

We also observed clear presence of virus‐positive cells in para‐bronchial glands (shown at two magnifications in Figure 3B), often in proximity to infected cartilage. Additional evidence of infection of both chondrocytes and para‐bronchial cells for additional patients is shown in supplementary material, Figure S4A and S4B, respectively. Syncytial cells also scored positive for the expression of spike (supplementary material, Figure S4C). In a minority of patients (5/27; 18.5%), we also detected sporadic spike positivity in the vascular wall, in both pericytes and endothelial cells (supplementary material, Figure S4D).

**Figure 4:**
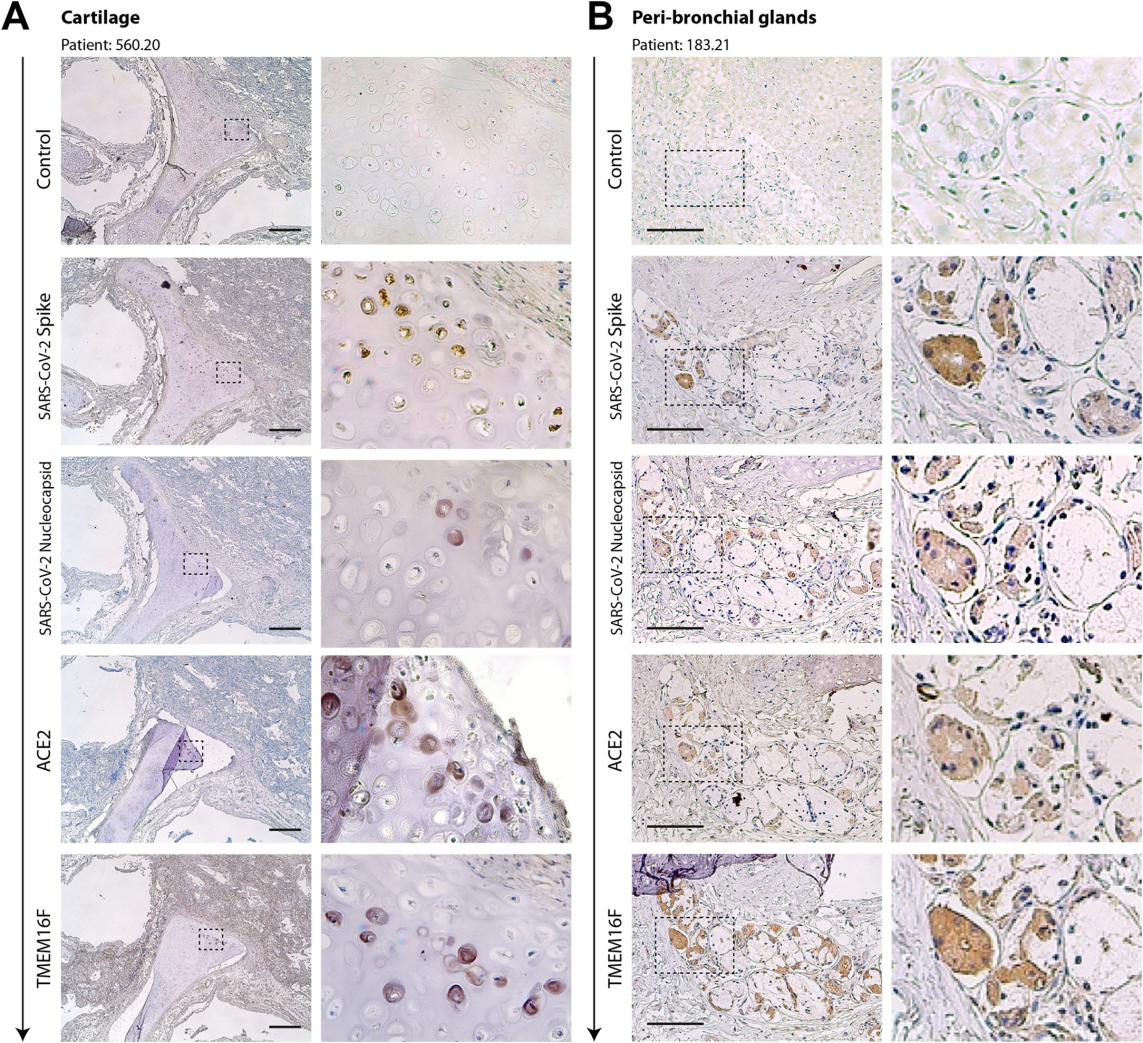
Serial sections of cartilage (A) and the peri‐bronchial area (B) of two former COVID‐19 patients. Serial sections (from top to bottom, arrow) from the same paraffin block were stained with antibodies against spike, N, ACE2, and TMEM16F, as indicated on the left of each picture. Representative pictures of cartilage and peri‐bronchial glands (panels A and B, respectively) are shown. For each picture, low‐ and high‐magnification images are shown; the magnified area is indicated by a dashed box. In most cases, positivity for immunodetection by all four antibodies (brown cells) was in superimposable regions. Scale bars: 100 μm.

We then analysed serial sections to assess whether spike and nucleocapsid proteins were localised in the same cells or anatomical structures. Figure 4 shows serial sections of a para‐bronchial region in patients 560.20 and 183.21, stained with both anti‐spike and anti‐nucleocapsid antibodies. A similar pattern of positivity was detected for these antigens in both chondrocytes and mucosal acinar gland cells (Figure 4A and 4B, respectively). In patient 560.20, we detected fibrous‐hyaline degeneration of the tracheal rings, with multiple pyknotic and dystrophic, virus‐positive cells. The same serial sections were also stained for the ACE2 viral receptor and the TMEM16F Ca^2+^ activated scramblase, which our work has shown to be activated by SARS‐CoV‐2 spike [5]. Both proteins were found to be expressed by both chondrocytes and mucous bronchial gland cells. This observation is consistent with the conclusion that SARS‐CoV‐2 directly infects and exerts pathological effects in these cells. SARS‐CoV‐2 infection [24, 25] and the presence of dystrophic alterations [4] in bronchial tissue were also reported previously in patients who died of acute COVID‐19. Positivity for both spike and nucleocapsid in patients with molecularly positive COVID‐19 is also shown in four patients from our Centre in supplementary material, Figure S5.

Finally, for 17 of the patients in our cohort, we could successfully extract RNA from formalin‐fixed, paraffin‐embedded blocks and test it for the presence of viral RNA by quantitative RT‐PCR by pooling ten sections from at least five specimens per patient. We used two different primer and probe sets, one specific for the SARS‐CoV‐2 nucleocapsid and the other for the ORF1ab genes (see Materials and methods). The number of positive samples for at least one amplicon was 9/17 (53%), of which five were positive for both amplicons. The detected viral RNA copy number for ORF1ab, which was the most sensitive assay, is reported in Table 1.

## Discussion

Here, we analysed a cohort of patients who seemingly recovered from SARS‐CoV‐2 infection, as concluded from multiple negative PCR tests on both nasopharyngeal swabs and bronchoalveolar lavage protracting for up to 300 days, but who still clinically deteriorated and eventually died. In most of these cases, pneumonia was the primary or co‐primary cause of death, a finding that was confirmed by pathology examination of the lungs. Our work shows that these patients, despite the apparent molecular negativity, still harboured virus‐infected cells in their lungs, particularly in the para‐bronchial glands and in the bronchial cartilage. This is consistent with the conclusion that these patients had never cleared the infection. The absence of SARS‐CoV‐2 infection in the respiratory epithelium possibly explains the apparent negativity of these patients to PCR tests performed on bronchoalveolar lavage. These results highlight the relevance of post‐mortem pathology examination as a crucial diagnostic tool [26, 27, 28].

What is the nature of the virus in the lungs of these individuals who died long after their last PCR‐positive test? Infection appears to be essentially restricted to para‐bronchial glands and bronchial chondrocytes, with sporadic presence of virus‐infected cells in the vascular wall of medium‐sized or large pulmonary arteries, in which clusters of either endothelial cells or mural cells seem to be infected, as shown by the expression of both the spike and the nucleocapsid antigens. Current evidence indicates that the production of significant levels of infectious virus does not necessarily follow the persistence of cells harbouring viral genomes [29, 30]. The lack of infectious virus isolation has been explained by the presence of fragments of the virus RNA and/or the production of defective viral particles [31]. However, irrespective of whether infection is productive or not in terms of new viral particle generation, the virus that we detect in these patients is translationally active, as it generates spike and N antigens that can be detected by antibody‐based techniques. Thus, the persistence of these virus‐infected cells can induce pathology itself, and also prolong abnormal stimulation of immune reactivity. In particular, the presence of the spike protein on the cell surface could have pathogenic relevance. Our previous work has shown that spike activates the cellular Ca^2+^‐dependent chloride channel and scramblase TMEM16F, thus promoting the externalisation of phosphatidylserine (PS) onto the outer leaflet of the cell plasma membrane and favouring cell–cell fusion [5]. PS externalisation is an important signal for monocyte/macrophage activation (reviewed in [32]) and is essential for the pro‐coagulant phase of platelet activation. In platelets, activated TMEM16F itself promotes lipid scrambling [33, 34], and externalised PS serves as an anchoring site for the assembly of the tenase and prothrombinase complex, which jointly enhance the rate of thrombin generation by several orders of magnitude [35]. Our more recent work indeed shows that cells expressing spike on their surface can directly activate TMEM16F in platelets and promote a pro‐coagulant phenotype (bioRxiv doi: 10.1101/2021.12.14.472668). Finally, spike‐expressing cells also impair the lymphocyte response by inducing fusion of these cells with SARS‐CoV‐2‐infected cells [36]. Thus, the prolonged presence of virus‐infected cells in these patients could maintain a pro‐inflammatory and pro‐thrombogenic status, independent of viral shedding, which might have resulted in the progressive deterioration of lung function and death. Our observations indeed indicate that lung pathology in these patients was essentially no different from that of acutely infected COVID‐19 cases.

An important question is to understand whether our findings might have relevance in explaining the persisting symptomatology of individuals with the long COVID syndrome, which consists in either continuous or relapsing and remitting COVID‐19 symptoms in PCR‐negative subjects [37, 38, 39, 40]. Persistent symptoms in long COVID patients have been variously attributed to organ damage, chronic inflammation, autoimmunity, or co‐morbidities exacerbated by intervening SARS‐CoV‐2 infection [41]. Lack of complete clearance of viral infection at >100 days from apparent molecular remission has been documented in the transplanted lungs from an individual who otherwise scored negative to standard PCR tests [42] and in at least another case of an infectious lung transplant in an otherwise PCR‐negative donor [43].

There are a few limitations in our study. Given our relatively small sample size, we are not able to identify what are the clinical characteristics of those patients in whom viral clearance had not occurred. Viral persistence does not seem to correlate with the severity of the prior COVID‐19 disease or its duration before the patients became apparently negative, but this will require broader investigation. *In situ* hybridisation (ISH) for viral RNA would have strengthened our conclusions. However, we found that the post‐mortem samples that we had available were of low quality for ISH, with suboptimal RNA preservation (this could also explain the quantitative variation in RNA levels that we measured by qRT‐PCR).

Despite these limitations, our findings indicate that SARS‐CoV‐2 infection can persist significantly longer than suggested by PCR‐negative tests on nasopharyngeal swabs or bronchoalveolar lavage fluids. Whether the persisting infected cells have a pathogenic role in explaining the sequelae of infection in long COVID remains an outstanding question, which deserves further investigation.

## Author contributions statement

RB performed the post‐mortem study and histological analysis, and supervised the study. LZ supervised molecular studies. RC and AC performed RT‐PCR analysis. FS analysed histology. SZ supervised the study. CC performed immunohistochemistry and supervised the study. MG supervised the study and wrote the manuscript. All the authors approved the final version of the manuscript.

## Supporting information


**Table S1.** Additional characteristics of the former COVID‐19 patients reported in this study
**Figure S1.** Additional lung pathology findings in a former COVID‐19 patient
**Figure S2.** Negative controls for immunohistochemistry
**Figure S3.** Absence of SARS‐CoV‐2 infection in the respiratory epithelium of former COVID‐19 patients
**Figure S4.** Additional evidence of SARS‐CoV‐2 positivity in samples from previous COVID‐19 patients
**Figure S5.** Immunohistochemistry in acute COVID‐19 patients

## Data Availability

De‐identified patient data collected for the study will be made available by writing to MG (mauro.giacca@kcl.ac.uk) or RB (bussani@units.it).
