# Supplementary material for: Persistent SARS‐CoV‐2 infection in patients seemingly recovered from COVID‐19
Source: J Pathol. 2023 Jan 18;259(3):254–63. doi: 10.1002/path.6035 (PMC10107739; doi:10.1002/path.6035)
Supplement: Supplementary file 1 — Table S1. Additional characteristics of the former COVID‐19 patients reported in this study Figure S1. Additional lung pathology findings in a former COVID‐19 patient Figure S2. Negative controls for immunohistochemistry Figure S3. Absence of SARS‐CoV‐2 infection in the respiratory epithelium of former COVID‐19 patients Figure S4. Additional evidence of SARS‐CoV‐2 positivity in samples from previous COVID‐19 patients Figure S5. Immunohistochemistry in acute COVID‐19 patients [file PATH-259-254-s001.docx]

**Persistent SARS-CoV-2 infection in patients seemingly recovered from COVID-19**

R Bussani *et al.* *J Pathol* <https://doi.org/10.1002/path.6035>

**Supplementary Table S1**

**Supplementary Figures S1–S5**

**Table S1. Additional characteristics of the former COVID-19 patients reported in this study.**

| Patient code | Comorbidities | Cause of final hospital admission | Therapies before hospital admission | Co-infections | End-stage extreme therapies |
| --- | --- | --- | --- | --- | --- |
| AUT. 256.20 | Heart failure | Femur fracture | Not known |  |  |
| AUT. 314.20 | Decubitus ulcers, erosive gastropathy, rectal ulcers, heart failure | Rectal bleeding | Not known |  |  |
| AUT. 327.20 | Decubitus ulcers, Parkinson’s disease, nephroangiosclerosis, pulmonary artery thrombosis | Acute respiratory failure, dehydration | Benserazide, enoxaparin |  |  |
| AUT. 448.20 | Sepsis, atrial fibrillation, dementia | Gangrenous cholecystitis | Quetiapinen, trazodone, amiodarone |  |  |
| AUT. 560.20 | Tracheomalacia, empyema, sepsis, bilateral pneumonia, decubitus ulcers | COVID-related acute respiratory failure | Not known | *Staphylococcus aureus* | Tracheostomy |
| AUT. 614.20 | Multiple hepatic abscesses, heart failure, previous stroke, diabetes, pneumonia | Diarrhoea, severe anaemia | Quetiapine, trazodone, clopidogrel, ramipril, finasteride, lansoprazole, rosuvastatin, tamsulosin, enoxaparin, metformin | *S. aureus* |  |
| AUT. 650.20 | Chronic lymphocytic leukaemia, decubitus ulcers, pyelonephritis | Urosepsis | Aloperidol, nitrofurantoin, enoxaparin | *Enterobacteriaceae* |  |
| AUT. 780.20 | Diverticulitis, cholaemic nephrosis | Decompensated liver cirrhosis, pleural effusion | Furosemide, vitamin K | *S. aureus* |  |
| AUT. 830.20 | Renal dysplasia, atrial fibrillation | Heart failure, pulmonary sepsis, pleural effusion | Spironolactone, bisoprolol, furosemide, acenocoumarol, tiotropium, metolazone |  |  |
| AUT. 831.20 | Hypertension | Acute limb ischaemia | Furosemide, lansoprazole, lysine acetylsalicylate, amiloride, spironolactone, triazolam |  |  |
| AUT. 809.20 | Heart failure, hypertensions, atrial fibrillation, pleural plaques, lung calcifications, pneumonia | COVID-related acute respiratory failure | Sotalol, warfarin, spironolactone, amlodipine, doxazosin, clonidine, furosemide, levothyroxine, lansoprazole |  |  |
| AUT. 692.20 | Femur fracture | Heart failure, acute aspiration pneumonitis | Aspirin, bromazepam, risperidone, timolol |  |  |
| AUT. 850.20 | Liver cirrhosis, pneumonia | COVID-related acute respiratory failure | Furosemide, vitamin K | *Candida albicans* | Tracheostomy |
| AUT. 857.20 | Diabetes, hypertension, COPD, erysipelas, MGUS | Heart failure | Bisoprolol, furosemide, fluticasone, aspirin, gliclazide |  |  |
| AUT. 41.21 | Pneumothorax | COVID-related acute respiratory failure | Not known |  | Tracheostomy, ECMO |
| AUT. 52.21 | Lung cancer with pleural and lymph node metastasis, resected laryngeal cancer, COPD, PAD | COVID-related acute respiratory failure | Tramadol, dexketoprofen, hyoscine butylbromide, bisphosphonate |  |  |
| AUT. 108.21 | Extraperitoneal haematoma of the pelvis | COVID-related acute respiratory failure | Not known |  | Tracheostomy |
| AUT. 144.21 | Hypertension, MI, PAD, dementia | Acute renal failure, dehydration, hypernatremia | Amlodipine, vitamin D, lorazepam, tramadol, enoxaparin |  |  |
| AUT. 145.21 | COVID-related pneumonia | COVID-related acute respiratory failure | Bisoprolol, fluticasone | *C. albicans*, HSV, *Aspergillus* | Tracheostomy, ECMO |
| AUT. 165.21 | Diabetes, hypertensions, liver steatosis, pulmonary calcifications | Sepsis, bilateral pneumonia | Ramipril, enoxaparin, propafenone, bisoprolol |  |  |
| AUT. 183.21 | COVID-related pneumonia | COVID-related acute respiratory failure | Not known | *Morganella morganii*, *Serratia* *marcescens*, CMV | Tracheostomy, ECMO |
| AUT. 208.21 | Lung carcinoma, mitral valve regurgitation, hypertension, dementia | Respiratory failure, advanced lung cancer | Vitamin B12, ramipril, tiotropium |  |  |
| AUT. 223.21 | Diabetes, AF, COPD, pleural calcification, asbestosis, cardiomyopathy, PAD | Heart failure | Tiotropium, salmeterol/fluticasone, bisoprolol, ambroxol, tamsulosin |  |  |
| AUT. 823.20 | Pneumonia, Alzheimer’s disease, bilateral suppurative nephritis | Trauma | Not known | *Enterobacteriaceae* |  |
| AUT. 237.21 | COVID-related pneumonia | COVID-related acute respiratory failure | Not known | *S. aureus*, *C. albicans* | Tracheostomy, ECMO |
| AUT. 299.21 | Hypertension, lung carcinoid | COVID-related acute respiratory failure | Telmisartan | *Enterococcus*, *C. albicans*, *C. glabrata*, *S. aureus*, *Enterobacteriaceae*, CMV, EBV, HSV, *Aspergillus* | Tracheostomy |
| AUT. 315.21 | Age-related macular degeneration, thrombocytopenia, hypertension, phlegmon of the hip | COVID-related acute respiratory failure | Bisoprolol, folic acid | *S. aureus* |  |

COPD, chronic obstructive pulmonary disease; ECMO, extracorporeal membrane oxygenation; AF, atrial fibrillation; HSV, herpes simplex virus; MGUS, monoclonal gammopathy of undetermined significance; MI, myocardial infarction; PAD, peripheral arterial disease.

**Supplementary Figures S1–S5**

**
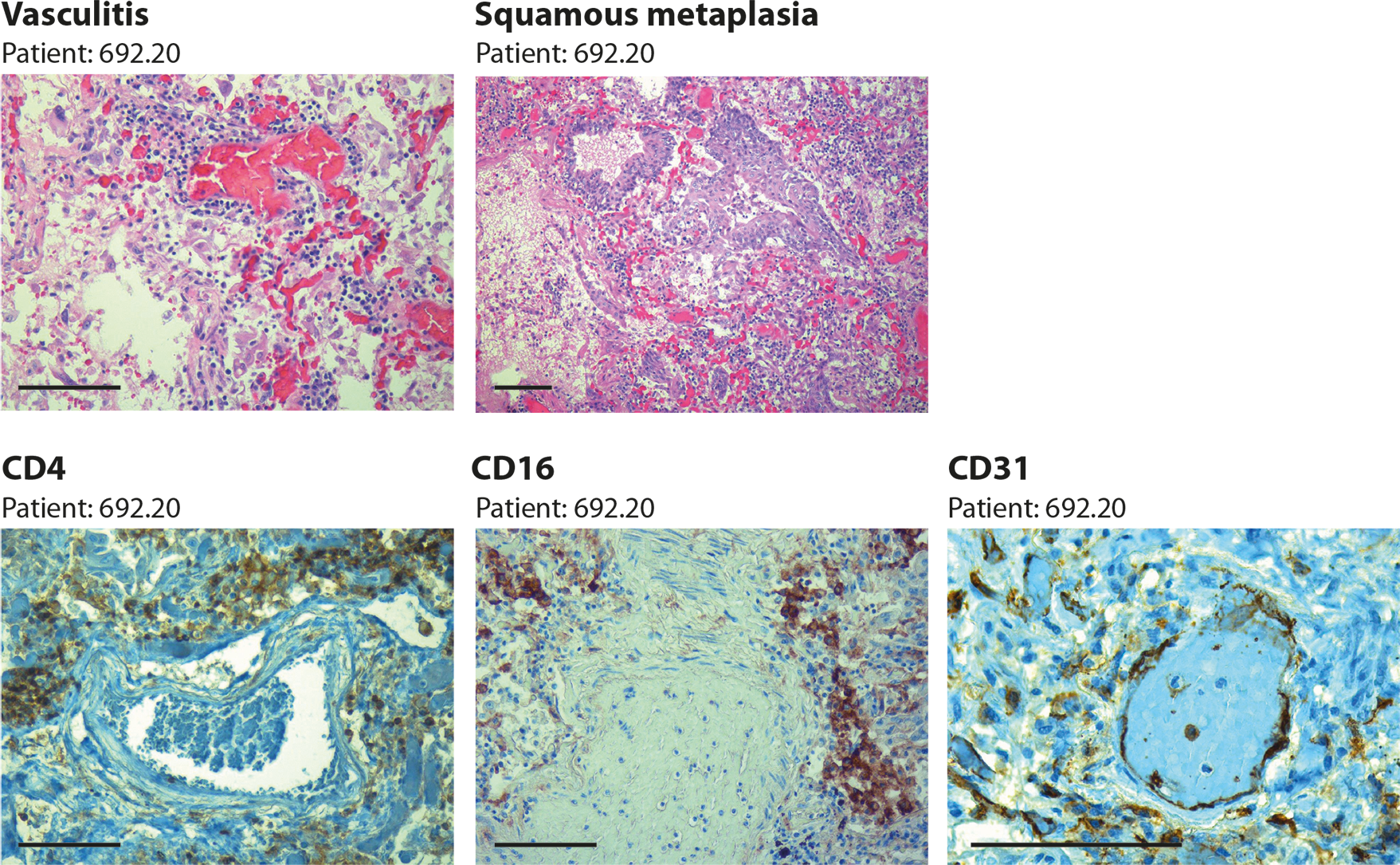
**

**Figure S1. Additional lung pathology findings in a former COVID-19 patient.** The top two pictures show representative images for areas with vasculitis and squamous metaplasia. The bottom three pictures show immunoreactivity of inflamed vessels for perivascular CD4 and CD16 cells, along with the presence of CD31 exfoliated epithelium.


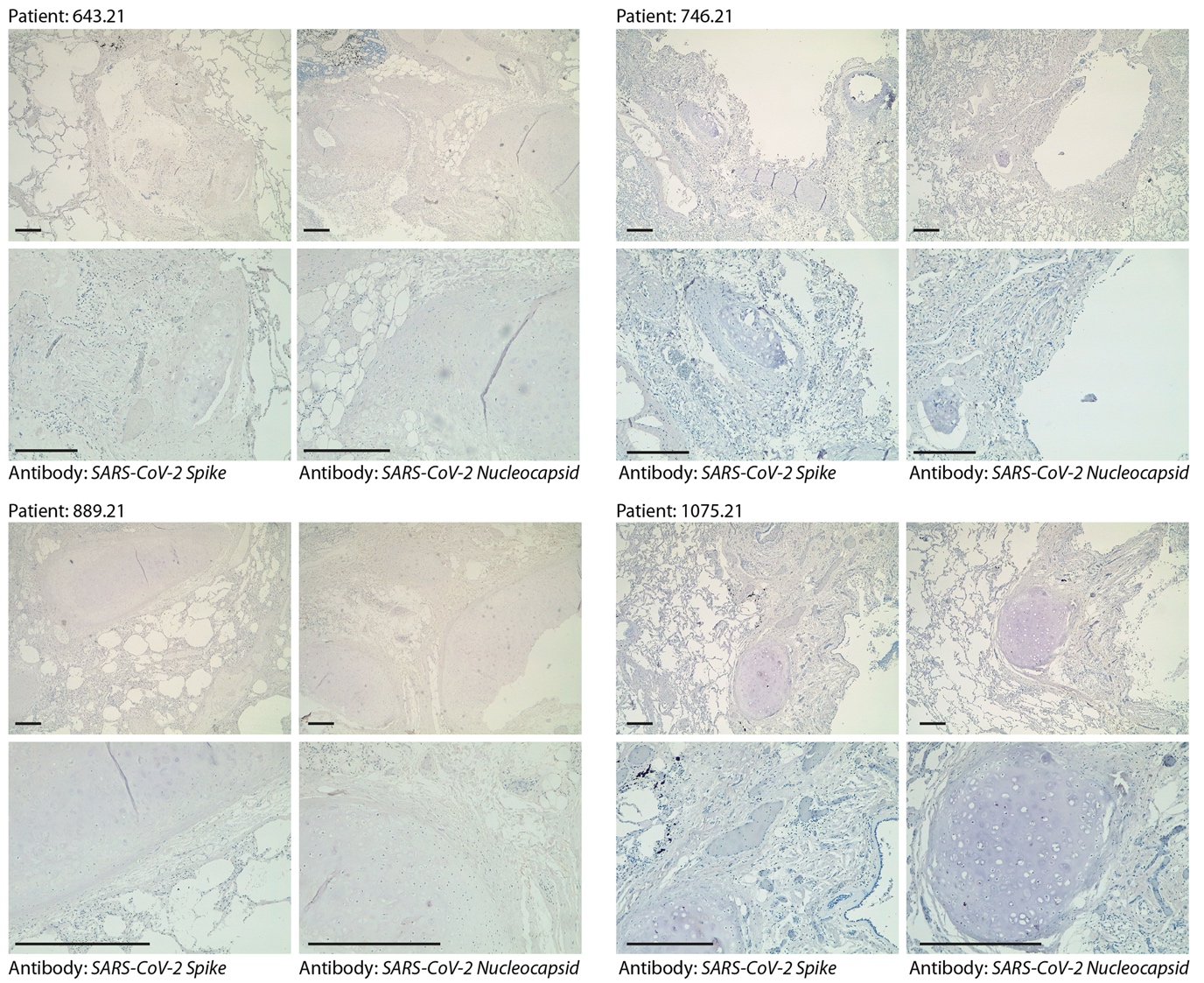


**Figure S2. Negative controls for immunohistochemistry.** The figure shows representative images of IHC assays using antibodies against the spike and nucleocapsid (N) antigens, as indicated, in samples from four different patients who died of COVID-19-unrelated conditions. No positivity is detectable in any structure. Scale bars in all panels: 100 µm.


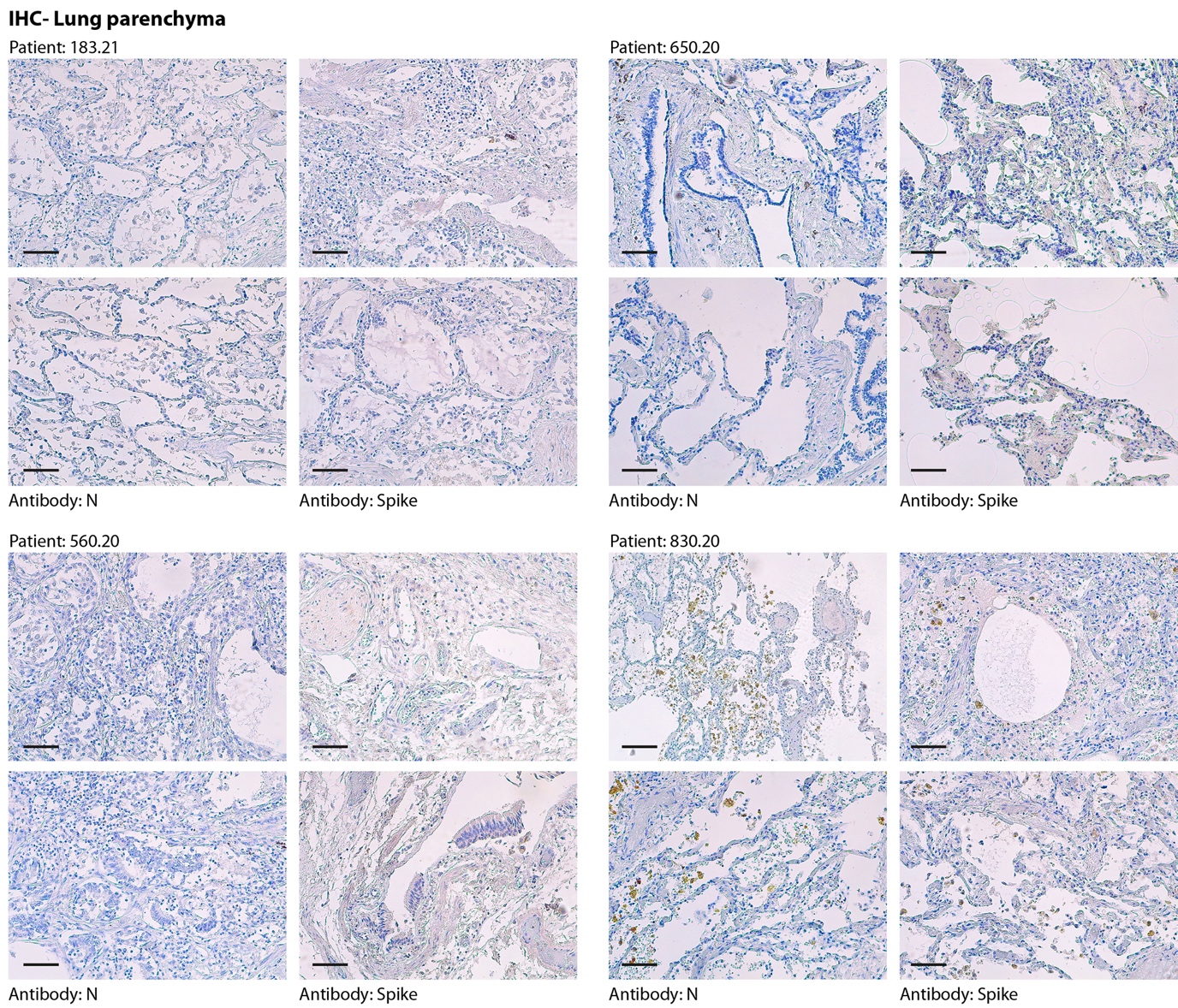


**Figure S3. Absence of SARS-CoV-2 infection in the respiratory epithelium of former COVID-19 patients.** The figure shows the results of immunohistochemistry (IHC) assays using antibodies against the spike and nucleocapsid (N) antigens in samples from four different previous COVID-19 patients (two samples per patient), as indicated at the top of each group of pictures. No positivity for SARS-CoV-2 was detectable in most part of the bronchial and alveolar epithelium in these patients. Scale bars: 100 µm.


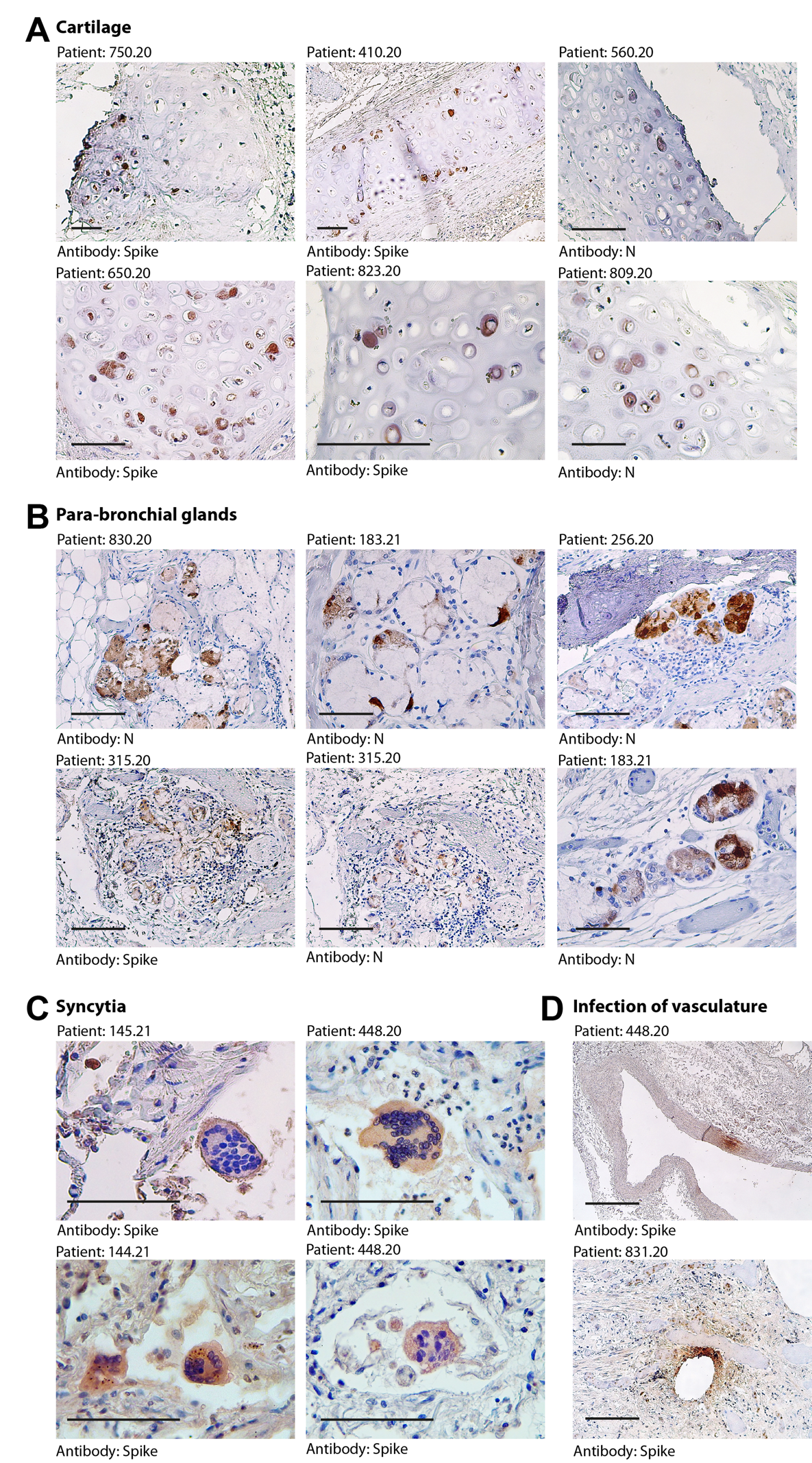


**Figure S4. Additional evidence of SARS-CoV-2 positivity in samples from previous COVID-19 patients.** The pictures show additional results from immunohistochemistry using antibodies against the SARS-CoV-2 spike and nucleocapsid (N) antigens in samples from different previous COVID-19 patients, as indicated at the top of each picture. Scale bars in all panels: 100 µm. **(A)** Samples from cartilage. **(B)** Samples from the peri-bronchial area, showing positivity in para-bronchial glands. **(C)** Spike-positive syncytial cells. **(D)** Sporadic spike positivity in perivascular cells of pulmonary arteries.


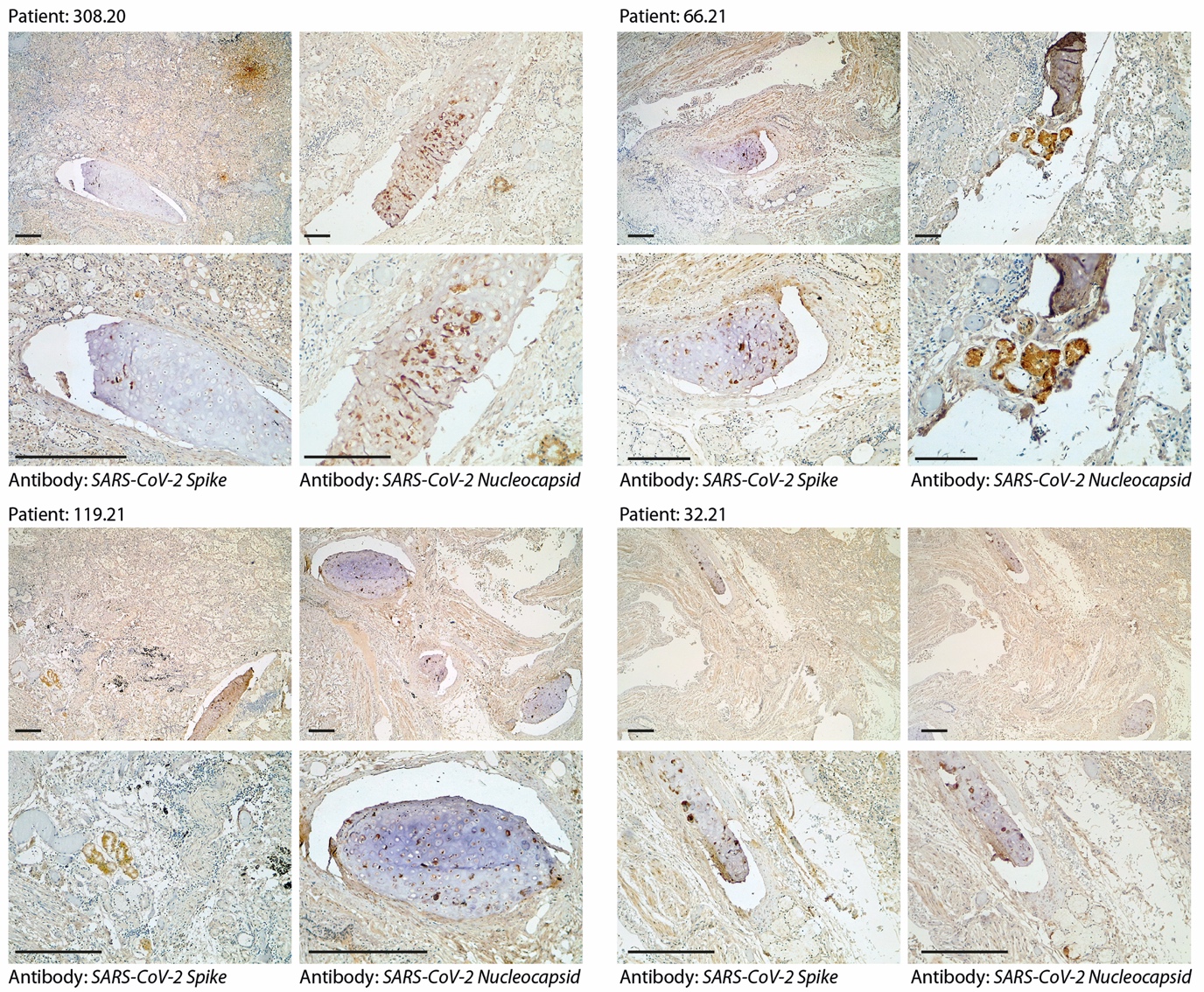


**Figure S5. Immunohistochemistry in acute COVID-19 patients.** The figure shows representative images of IHC assays using antibodies against the spike and nucleocapsid (N) antigens, as indicated, in samples from four different patients who died of COVID-19. SARS-CoV-2-positive cells are clearly detectable in bronchial cartilage chondrocytes and in several mucosal glands proximal to the bronchial structures. Scale bars in all panels: 100 µm.
